# Analysis of Bone Phenotype Differences in MEN1-Related and Sporadic Primary Hyperparathyroidism Using 3D-DXA

**DOI:** 10.3390/jcm13216382

**Published:** 2024-10-25

**Authors:** Anna K. Eremkina, Svetlana V. Pylina, Alina R. Elfimova, Anna M. Gorbacheva, Ludovic Humbert, Mirella López Picazo, Angelina V. Hajrieva, Ekaterina N. Solodovnikova, Liliya D. Kovalevich, Ekaterina A. Vetchinkina, Ekaterina V. Bondarenko, Natalia V. Tarbaeva, Natalia G. Mokrysheva

**Affiliations:** 1 Endocrinology Research Centre, 115478 Moscow, Russia; pylina.svetlana@endocrincentr.ru (S.V.P.); ainetdinova.alina@endocrincentr.ru (A.R.E.); gorbacheva.anna@endocrincentr.ru (A.M.G.); hayrieva.angelina@endocrincentr.ru (A.V.H.); solodovnikova.ekaterina@endocrincentr.ru (E.N.S.); kovalevich.liliya@endocrincentr.ru (L.D.K.); vetchinkina.katya@endocrincentr.ru (E.A.V.); bondarenko.ekaterina@endocrincentr.ru (E.V.B.); tarbaeva.natalya@endocrincentr.ru (N.V.T.); mokrisheva.natalia@endocrincentr.ru (N.G.M.); 2 3D-Shaper Medical, 08007 Barcelona, Spain; ludovic.humbert@3d-shaper.com (L.H.); mirella.lopez@3d-shaper.com (M.L.P.)

**Keywords:** MEN1-related primary hyperparathyroidism, multiple endocrine neoplasia type 1, *MEN1*, BMD, 3D-DXA

## Abstract

**Background:** The rarity and variability of MEN1-related primary hyperparathyroidism (mPHPT) has led to contradictory data regarding the bone phenotype in this patient population. **Methods:** A single-center retrospective study was conducted among young age- and sex-matched patients with mPHPT and sporadic hyperparathyroidism (sPHPT). The main parameters of calcium–phosphorus metabolism, bone remodeling markers, and bone mineral density (BMD) measurements were obtained during the active phase of hyperparathyroidism before parathyroidectomy (PTE) and 1 year after. Trabecular Bone Score (TBS) and 3D-DXA analysis of the proximal femur were used to evaluate the differences in bone architecture disruption between groups. **Results:** Patients with mPHPT had significant lower preoperative BMD compared to sPHPT at lumbar spine—LS (*p* = 0.002); femur neck—FN (*p* = 0.001); and total hip—TH (*p* = 0.002). 3D-DXA analysis showed the prevalence of cortical rather than trabecular bone damage in mPHPT compared to sPHPT: cortical thickness (*p* < 0.001); cortical surface BMD (*p* = 0.001); cortical volumetric BMD (*p* = 0.007); and trabecular volumetric BMD (*p* = 0.029). One year after, PTE DXA and 3D-DXA parameters were similar between groups, while 3D-visualisation showed more extensive regeneration in cortical sBMD and cortical thickness in mPHPT. **Conclusions:** mPHPT is associated with lower preoperative BMD values with predominant architecture disruption in the cortical bone. The absence of differences in DXA and 3D-DXA parameters 1 year after PTE between mPHPT/sPHPT combined with significantly lower BMD in mPHPT at the initial stage may indicate faster bone recovery after surgery in mPHPT than in sPHPT.

## 1. Introduction

Multiple endocrine neoplasia type 1 (MEN1) is a rare autosomal dominant hereditary syndrome caused by a germline mutation in the *MEN1* gene and characterized by a predisposition to various endocrine and non-endocrine tumors [1]. The most common manifestations of MEN1 syndrome are primary hyperparathyroidism (PHPT), duodeno-pancreatic neuroendocrine neoplasms (NENs), and pituitary adenomas [2]. MEN1-related PHPT (mPHPT) is estimated to account for approximately 2–4% of all PHPT cases. It is the first clinical manifestation of the disease in approximately 90% of patients, with a median age of onset between 20 and 25 years [3]. The main differences between mPHPT and the sporadic disease (sPHPT) include: earlier age of onset, equal sex ratio, multiglandular parathyroid involvement (more often hyperplasia), family history, and associated NENs. In most cases, mPHPT can remain asymptomatic for a long time, but mild to moderate progression to hypercalcaemia typically begins in adolescence, and almost all MEN1 patients are expected to have hypercalcemia by the age of 50 [4]. The term “asymptomatic”, however, is open to debate and does not necessarily imply only laboratory values. The nonspecific symptoms of parathyroid hormone (PTH) elevation may remain undetected. Data on PHPT-associated complications in MEN1 patients remain controversial. Twigt et al. found no significant differences in renal complications between sPHPT and mPHPT [5]. In contrast, Lourenco DM et al. reported the higher frequency and earlier onset of the urolithiasis (up to 86.2% in individuals under 30 years of age) in the MEN1 group [6]. It is considered that bone involvement is more severe in MEN1 compared to sPHPT. A greater decrease in bone mineral density (BMD) at the lumbar spine and femoral neck was observed in patients with mPHPT compared to sPHPT [7,8]. According to Silva et al., the rate of bone formation after parathyroidectomy (PTE) is significantly lower in mPHPT than in sPHPT [9]. BMD analysis of the three major bone sites—spine, radius, and hip—showed early, severe, and frequent bone demineralization in patients with mPHPT [10]. Presumably, these differences may be related to earlier loss of BMD associated with earlier disease onset (approximately three decades before sPHPT), and longer exposure to elevated PTH concentrations, which may have a negative impact on the acquisition of peak bone mass. Other MEN1-related manifestations such as hypercortisolism, hyperprolactinemia, hypogonadism, growth hormone deficiency, and/or nutrient malabsorption caused by NENs therapy may exacerbate bone loss in mPHPT patients [11]. Thus, recently Altieri et al. [12] demonstrated a high incidence of osteopenia and osteoporosis in patients with MEN1 and gastrointestinal NENs. The main causes were nutritional deficiencies due to hypersecretion of gastrointestinal hormones, therapy with somatostatin analogues and/or chemotherapy, and malabsorption after extensive surgery of the duodenum and pancreas. Despite advances in the management of MEN1-related tumors, limited genetic screening results in diagnostic delay for young patients with mPHPT who do not meet the clinical and familial criteria for this syndrome, which can worsen the prognosis of bone disease [4]. In addition, it is unknown which type of bone tissue (cortical or trabecular) is predominantly involved in mPHPT and what the rate of BMD recovery after PTE is.

## 2. Materials and Methods

### 2.1. Study Design

**Figure 1:**
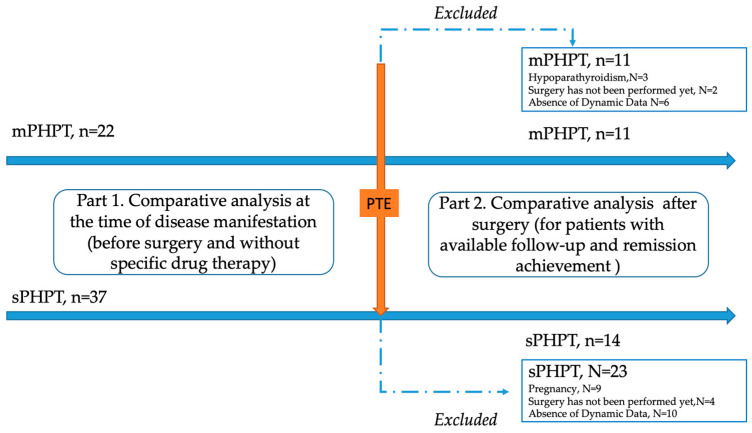
Flow chart of the included patients with MEN1-related (mPHPT) and sporadic hyperparathyroidism (sPHPT). PTE—parathyroidectomy.

A single-center retrospective study was conducted at the Endocrinology Research Centre, Moscow (ERC) from 13 July 2017 to 30 December 2023. The genetically approved target population of mPHPT (N = 22) was defined according to the inclusion criteria: age over 18 years, confirmed PHPT according to the Russian guidelines, the presence of the *MEN1* gene or target gene panel sequencing, and the availability of laboratory and instrumental examination at the time of PHPT manifestation and 1 year after surgery. The comparison group (N = 37) included age- and sex-matched individuals with sPHPT without a mutation in the MEN1 gene with available laboratory and instrumental examination at the time of PHPT manifestation and 1 year after surgery. The exclusion criteria for both groups were: lack of sufficient data on the clinical course of PHPT (including bone complications and DXA results); data on previously performed PTE in medical history; menopause in women and age over 50 in men; severe and chronic diseases (cerebrovascular disease, ischemic heart disease, cardiac, respiratory, or hepatic insufficiency, cancer); diabètes mellitus type 1 or 2; and an estimated glomerular filtrate rate (eGFR) < 60 mL/min/1.73 m^2^). To exclude the influence of other endocrine disorders on the bone phenotype, additional exclusion criteria were thyrotoxicosis, hypothyroidism, and hypercortisolism. The detailed study design is shown in Figure 1.

### 2.2. Biochemical Evaluation

Patients underwent several laboratory tests outlined below. The ARCHITECT c8000 chemistry analyzer (Abbott, Abbott Park, IL, USA) was used to determine the concentrations of serum calcium (reference interval (RI) 2.15–2.55 mmol/L); albumin (RI 34–48 g/L female, RI 35–50 g/L male); phosphorus—(RI 0.74–1.52 mmol/L); creatinine (RI 50–98 µmol/L female, 63–110 µmol/L male); and 24-h urine calcium (RI 2.5–8.0 mmol/L). PTH (range 15–65 pg/mL), osteocalcin (range 11–43 ng/mL female, 14–42 ng/mL male) and b-CrossLaps (range 0.3–1.1 ng/mL female, range 0.1–0.85 ng/mL male) were measured on the COBAS 6000 (Roche, Rotkreuz, Switzerland). Albumin-corrected calcium was calculated using the formula: albumin-corrected calcium (mmol/L) = measured plasma calcium level (mmol/L) + 0.02 × (40 − albumin level (g/L)). eGFR was calculated using the CKD-EPI 2009 formula. Thyroid-stimulating hormone (TSH) (RI 0.25–3.5 mIU/L, ARCHITECT I2000SR (Abbott, Abbott Park, IL, USA) and glucose (RI 3.1–6.1 mmol/L, ARCHITECT c8000 (Abbott, Abbott Park, IL, USA) assays were used to exclude patients with thyroid, adrenal, and carbohydrate disorders. The oral glucose tolerance test was performed according to the standard algorithm if the glucose value was outside the RI. In the presence of an adrenal incidentaloma or clinical signs of hypercortisolism, an overnight suppression test with 1 mg dexamethasone was performed (a serum cortisol suppression of 50 nmol/L was used as the post-test cut-off point). The following additional parameters were also analyzed to exclude other MEN1 components: Insulin-like growth factor 1 (IGF1) (RI: 51–271 ng/mL female; 62–230 ng/mL male; LIAISON XL, DiaSorin, Saluggia, Italy), if necessary—growth hormone suppression test was measured (RI 0.06–6.9 ng/mL female; 0.02–1. 23 ng/mL male; LIAISON XL (DiaSorin, Saluggia, Italy); prolactin (RI 102–496 mU/L female; 86–324 mU/L male COBAS 6000 (Roche, Rotkreuz, Switzerland)—in the presence of clinical signs of hyperprolactinemia, menstrual dysfunction, and/or evidence of a pituitary incidentaloma. Chromogranin A (RI < 3 nmol/L; ELISA, DiaSourse Diagnostics, Louvain-la-Neuve, Belgium) and gastrin (RI 13–115 pg/mL, analysis at the Centre for Molecular Diagnostics, by the IHLA method) were assessed in patients with gastrointestinal NENs according to contrast enhanced computed tomography (CECT).

### 2.3. Instrumental Diagnosis

Preoperative imaging included ultrasonography of the parathyroid glands (Voluson E8, GE, Chicago, IL, USA) and technetium-99 sestamibi scintigraphy (Discovery NM/CT 670 SPECT/CT system, GE, Chicago, IL, USA). Screening for other components of MEN1 consisted of MSCT (Revolution CT; Optima CT, GE, Chicago, IL, USA) of the abdominal cavity and retroperitoneal space and MRI of the brain (SIGNA Pioneer, GE, Chicago, IL, USA), with intravenous contrast enhancement if necessary).

### 2.4. Screening for PHPT Complications

Patients underwent dual-energy X-ray absorptiometry (DXA) scans of the lumbar spine (LS) with assessment of trabecular bone score (RI > 1.310) (TBS), femoral neck (FN), total femur (TH), total radius (RT), and distal third of radius (R33%). DXA analyses were performed using Lunar iDXA enCORE GE Healthcare version 17; USA (TBS was measured by applying TBS iNsight software version 3.0.2.0) and Lunar iDXA enCORE GE Healthcare version 15; USA (TBS was measured by applying TBS iNsight software version 3.0.0.0); X-ray of thoracic and LS (X-ray diagnostic system Optima RF420, GE Healthcare, Chicago, IL, USA); renal ultrasound (Aplio 500, Toshiba, Tokyo, Japan) and/or renal CT scan (Optima CT660, GE, Chicago, IL, USA); and esophagogastroduodenoscopy (Olympus GIF-XP 150N, Olympus Corporation, Tokyo, Japan). Z-scores less than −2.0 SD were defined as BMD below the expected age ranges according to the International Society for Clinical Densitometry (ISCD).

### 2.5. 3D-DXA

3D-DXA advanced analysis of the DXA scans was performed using 3D-Shaper software (version 2.12.1 3D-shaper Medical, Barcelona, Spain). The following parameters of the proximal femur were assessed before and after surgery: cortical surface bone mineral density (cortical sBMD), mg/cm^2^; cortical volumetric bone mineral density (cortical vBMD), mg/cm^3^); cortical thickness, mm; and trabecular volumetric bone mineral density (trabecular vBMD, mg/cm^3^). 3D-DXA parameters were calculated at the TH and FN. The anatomical distribution of average differences in cortical thickness, vBMD, and sBMD between groups is shown on the periosteal surface of the femur using 3D visualization. Differences in cortical and trabecular vBMD are shown on cross-sectional images: mid-coronal; neck (the section perpendicular to the femoral neck axis with the minimal area); intertrochanteric (the mid-section of the intertrochanteric region); and lower shaft (the section in the proximal femur shaft).

### 2.6. Genetic Analysis

Genomic DNA was isolated from peripheral blood lymphocytes using the AllSheng Auto-Pure 96 Nucleic Acid Extraction System DNA RNA and NucleoSpin96 Blood Kit. DNA was quantified and analysed using a BioSpectrometer spectrophotometer (Eppendorf, Hamburg, Germany). The customized gene panel including coding region genes associated with hyperparathyroidism was applied. Sequencing was performed on the Illumina Miseq platform in 4 (out of 22) patients from the mPHPT group and 13 (out of 33) from the sPHPT group (*AIP*, *AP2S1*, *CASR*, *CDC73*, *CDKN1A*, *CDKN1B*, *CDKN1C*, *CDKN2A*, *CDKN2C*, *CDKN2D*, *DICER1*, *FAM111A*, *GATA3*, *GCM2*, *GNA11*, *GNAS*, *MEN1*, *POU1F1*, *PRKAR1A*, *PRKCA*, *PTEN*, *PTTG2*, *SDHA*, *SDHB*, *SDHC*, *SDHD*, *TBCE*). In the remaining patients, the *MEN1* gene (comprising 2–9 exons) was sequenced by the Sanger method using a Genetic Analyzer 3500 (Thermo Scientific, Waltham, MA, USA). The identified variants were described using reference sequencing NM_001370259.2. Clinical significance was determined using the guidelines of American College of Medicine genetic and Genomics (ACMG) [13]. An automated algorithm that aligns the reads to the human genome reference sequence (hg38) was used to process the sequencing data.

### 2.7. Statistical Analysis

Statistical analysis was performed using Python 3.11.5 with the following libraries: scipy 1.11.1 and matplotlib 3.7.2. Descriptive statistics for quantitative characteristics are presented as medians and interquartile ranges Me [Q1; Q3]; descriptive statistics for qualitative characteristics are presented as absolute and relative frequencies (n (%)). The Mann–Whitney test (U-test) and the two-sided Fisher’s exact test (F-test) were used to compare two independent groups for quantitative and qualitative characteristics, respectively. The Wilcoxon test (W-test) was used to compare two dependent groups for quantitative characteristics. The critical significance level used to test statistical hypotheses was 0.05. The Benjamini–Hochberg procedure (*p*_0_-value) was used to correct for the problem of multiple comparisons: *p*-value < *p*_0_-value was interpreted as a statistically significant result.

## 3. Results

### 3.1. Patients’ General Characteristics

The median duration of PHPT was comparable in both groups: 1 [0; 2] year in the mPHPT group vs. 1 [0; 1] year in the sPHPT group (*p* = 0.967, U-test). The median age was 36 [28; 39] years in the mPHPT group and 34 [30; 38] years in the sPHPT group (*p* = 0.981, U-test). The male to female ratio in the mPHPT was 1:4.5 vs. 1:8.25 in the sporadic one (*p* = 0.719, F-test). There were no differences in the preoperative parameters of calcium–phosphorus metabolism or the bone remodeling markers (osteocalcin, beta-CrossLaps) between groups (Table 1). PHPT was the only hormone-producing tumor in 18 patients; the remaining four patients had prolactinoma in addition to PHPT (N = 4, all patients achieved normoprolactinemia on cabergoline therapy) and insulinoma (N = 1). In 18 patients (82%), mPHPT was the first manifestation of MEN1. Among the non-functional MEN1-related components, there were 2 cases of pituitary microadenoma, 12 cases of pancreatic NENs and 1 case of adrenal cortical nodular hyperplasia. In the sPHPT group, there was one case of non-functioning pituitary microadenoma.

The results of genetic testing revealed the presence of seven new variants of the *MEN1* gene mutations that had not been previously described. The clinical significance was determined to be unknown in three cases, with clinical presentation of isolated PHPT in two cases and PHPT + non-functioning NENs in one case. The detailed description of the variants is presented in Table 2.

**Figure 2:**
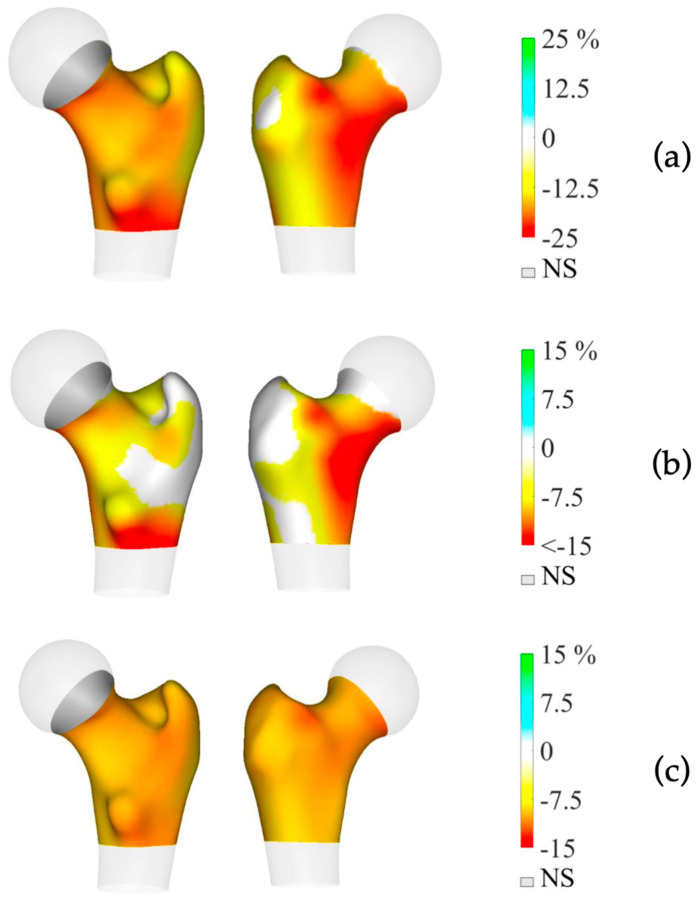
3D visualization of the percentage difference in cortical bone between mPHPT and sPHPT before parathyroidectomy. (**a**) Cortical sBMD; (**b**) Cortical thickness; (**c**) Cortical vBMD. Lower values of mPHPT compared to sPHPT are shown in yellow-red, no difference is shown in gray. mPHPT (MEN1-related primary hyperparathyroidism); sPHPT (sporadic primary hyperparathyroidism).

**Figure 3:**
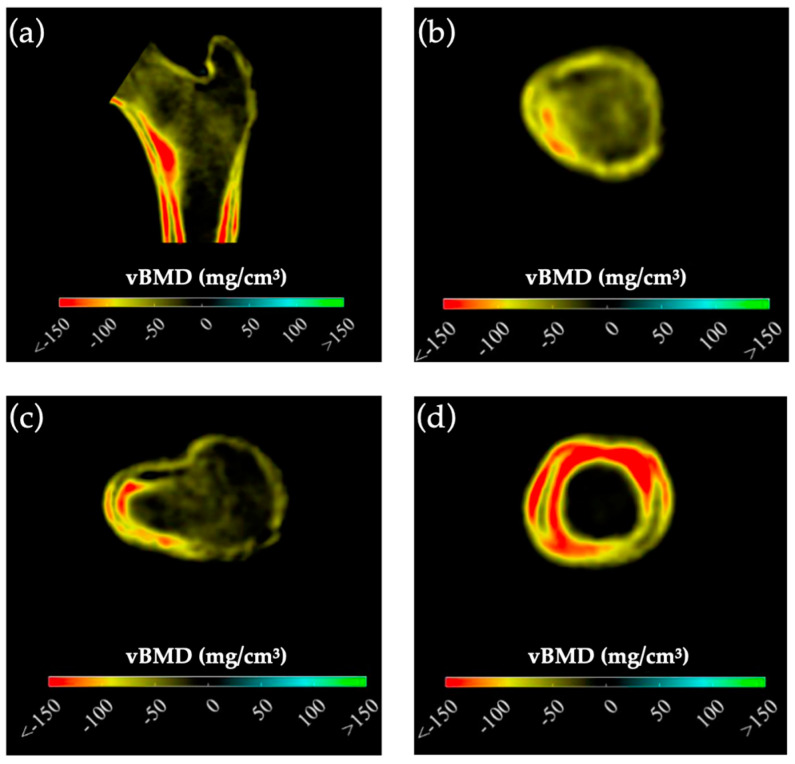
Cross-sectional images showing the difference in cortical and trabecular vBMD between mPHPT and sPHPT before surgery. Lower values of mPHPT compared to sPHPT are shown in yellow-red. (**a**) Mid-coronal section; (**b**) Neck section; (**c**) Intertrochanteric section; (**d**) Lower shaft section). mPHPT (MEN1-related primary hyperparathyroidism); sPHPT (sporadic primary hyperparathyroidism).

Preoperative BMD measurements showed statistically significant lower BMD in the mPHPT compared to the sPHPT at LS, FN, and TH. There were also statistically significant differences in Z-scores at the LS, FN, and R33% sites. 3D-DXA analysis showed lower values of cortical bone in mPHPT patients compared to sPHPT patients, whereas trabecular vBMD did not differ between groups TH (*p* = 0.029) and TBS (*p* = 0.136) (Table 3). 3D visualization of the percentage difference in cortical bone between groups showed more extensive damage in cortical sBMD and cortical thickness in mPHPT compared to sPHPT (Figure 2). The cross-sectional images show more extensive bone demineralization at the femur surface in the mPHPT group (Figure 3).

### 3.2. Follow-Up of Primary Hyperparathyroidism

In the mPHPT group, PTE was performed in 20 of 22 patients. A total of 10 patients underwent subtotal PTE (removal of 3 or 3.5 parathyroid glands); 7 patients underwent surgical removal of <3 parathyroid glands; and in 3 cases total PTE with autotransplantation (TPT + AT) was performed. In all cases, TPT + AT was associated with chronic hypoparathyroidism (N = 3). Histologic examination confirmed parathyroid adenomas in 12 cases, parathyroid hyperplasia in 3 cases and a combination of adenomas with diffuse nodular hyperplasia in 5 cases. In the sPHPT group, 33 patients underwent PTE. In all cases only one parathyroid gland was removed. Histological findings were adenomas, except for one case of atypical adenoma.

Dynamic follow-up data were available for 11 mPHPT and 14 sPHPT patients according to the inclusion and exclusion criteria. Dynamic follow-up was comparable in both groups: 1.2 [0.9; 1.3] years in mPHPT vs. 1.3 [1.1; 1.6] years in sPHPT (*p* = 0.267). A statistically significant increase in BMD was observed at all sites except the radius in both groups: BMD LS +8.5% for mPHPT (*p* = 0.008) and +2.5% for sPHPT (*p* = 0.005); BMD TH +2.1% for mPHPT (*p* = 0.005) and +9.6% for sPHPT (*p* = 0.002); BMD FN +4.3% for mPHPT (*p* = 0.007) and +6.2% for sPHPT (*p* = 0.002).

In the mPHPT group, 3D-DXA analysis showed a significant increase in all cortical and trabecular bone parameters at TH and FN compared to preoperative values (Table 4). The same result was observed in the sPHPT group except for cortical thickness, cortical sBMD FN, and trabecular vBMD TH (Table 5). Comparison of bone remodeling markers, DXA and 3D-DXA parameters between groups at 1 year after surgery did not reveal significant differences.

**Figure 4:**
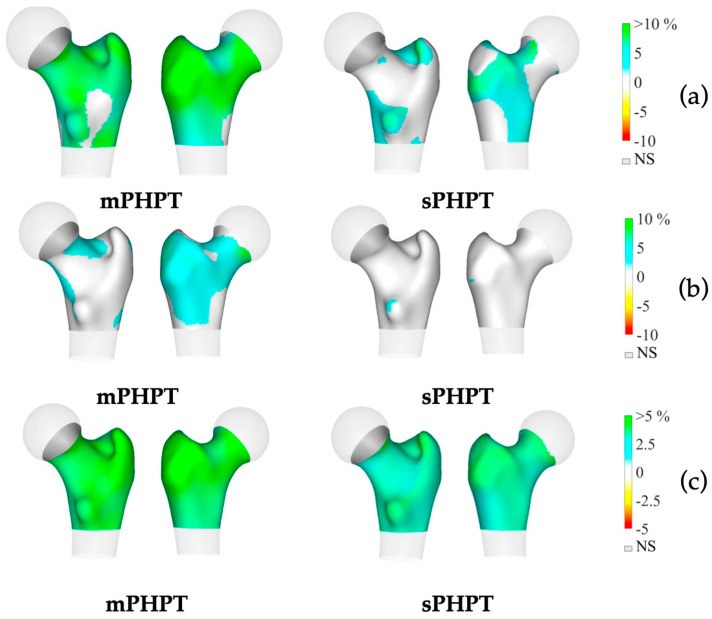
3D visualization of the percentage change in cortical bone in the mPHPT and sPHPT groups 1 year after parathyroidectomy. (**a**) Cortical sBMD; (**b**) Cortical thickness; (**c**) Cortical vBMD. Increase is shown in blue-green color, decrease is shown in yellow-red color, NS—no difference is shown in gray color. mPHPT (MEN1-related primary hyperparathyroidism); sPHPT (sporadic primary hyperparathyroidism).

**Figure 5:**
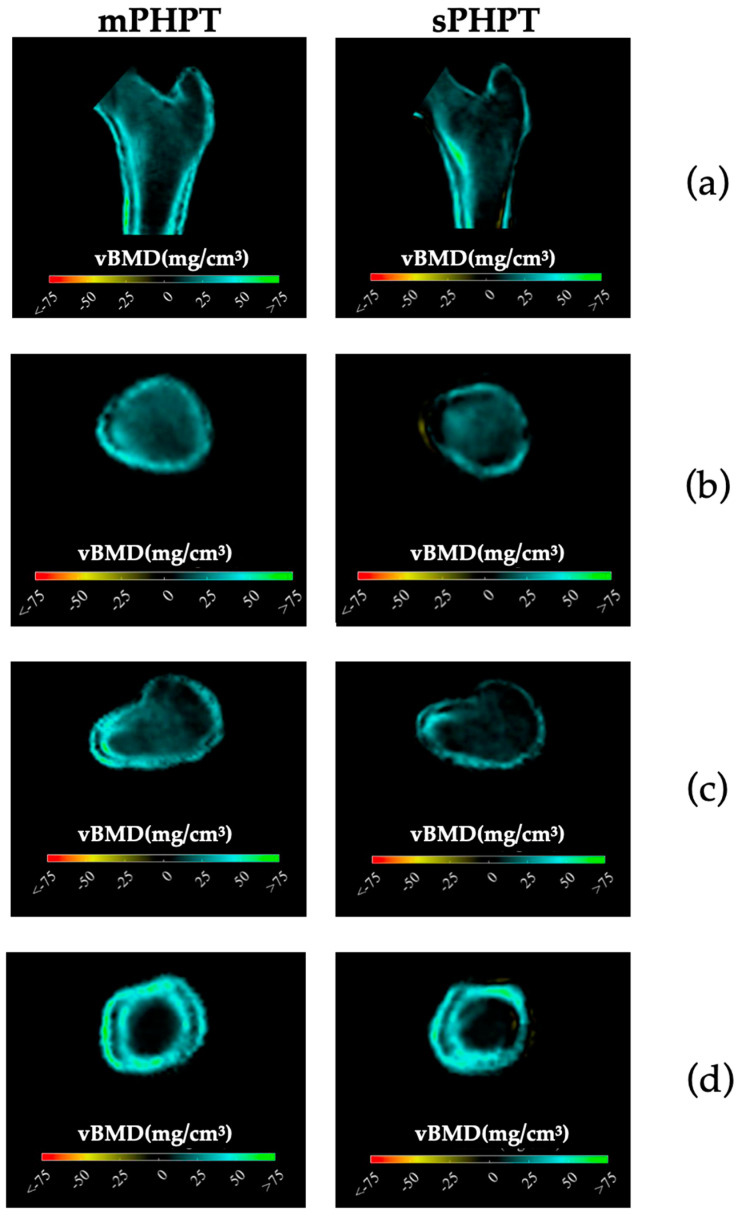
Cross-sectional images showing changes in cortical and trabecular vBMD in both groups 1 year after surgery (increase is shown in blue-green color, decrease is shown in yellow-red color, no difference is shown in black color). (**a**) Mid-coronal section; (**b**) Neck section; (**c**) Intertrochanteric section; (**d**) Lower shaft section. mPHPT (MEN1-related primary hyperparathyroidism); sPHPT (sporadic primary hyperparathyroidism).

3D-visualisation of percentage difference in cortical bone 1 year after PTE showed more extensive regeneration in cortical sBMD and cortical thickness in the mPHPT group, whereas in the sPHPT group, the increase in cortical thickness was not visually detectable (Figure 4). On cross-sectional images, vBMD recovery in cortical and trabecular bone was visually similar in both groups (Figure 5).

## 4. Discussion

The diagnosis of MEN1 is established if the patient has at least two of the classical manifestations of this syndrome (PHPT, a pituitary adenoma or NENs of gastro-entero-pancreatic tissue; clinical criteria) or at least one MEN1-related tumor and a first-degree relative with genetically confirmed MEN1 (familial criteria), or finally the pathologic mutation in the MEN1 gene in the index case (genetic criteria). Moreover, mPHPT is usually the first manifestation, can be isolated, and is not accompanied by multiglandular parathyroid involvement. Our study found that 81.8% (18/22) of patients had mPHPT as the only hormone-producing component of MEN1. This confirms the difficulties in using family and clinical criteria alone to diagnose MEN1 in young patients [4]. Genetic testing in all participants allowed us to avoid potential misclassification of patients between groups.

Data on the clinical and laboratory features in mPHPT and sPHPT are contradictory. According to current knowledge, mPHPT tends to have lower calcium, phosphorus, and PTH levels than sPHPT [4,5,38]. At the same time, Mathew et al. [8] demonstrated lower mean serum calcium in mPHPT patients, compared to sPHPT (*p* = 0.01), but PTH levels were comparable (*p* = 0.08). Our findings concur with those of the Russian PHPT Registry [39], indicating that patients with mPHPT exhibited no significant differences in PTH, phosphorus, and creatinine levels compared to patients with sPHPT.

The frequency of structural renal damage in both groups was similar (mPHPT 54.5% vs. sPHPT 62.2%, *p* = 0.594). Previously Kong et al. [40] showed that mPHPT patients are more prone to nephrolithiasis than sPHPT (60.0% vs. 40.2%, *p* = 0.024). Lourenco et al. [41] reported a higher prevalence of urolithiasis in the combination of mPHPT and gastrinoma (93.3%) rather than in mPHPT alone (71.4%, *p* = 0.106). According to Twigt et al., [5] the incidence of renal calculi was not significantly different between sPHPT, MEN1, and MEN2A patients (*p* = 0.184 and *p* = 0.06 versus *p* = 0.22 and *p* = 0.59, respectively).

Spine and hip were the most evaluated sites for both mPHPT and sPHPT. Some authors showed lower Z-scores for LS and FN in mPHPT vs. sPHPT, but this was not confirmed for absolute BMD values [7,8]. On the other hand, Kong et al. [40] found no difference of Z-score in LS (*p* = 0.928) and FN (*p* = 0.749) between mPHPT and sPHPT patients. In our research, mPHPT patients had significantly lower preoperative Z-scores and BMD values at LS, FN, and TH, which is inconsistent with Marini et al. [42]. According to this research, mPHPT and sPHPT were comparable in preoperative BMD at spine and hip, but patients with MEN1 were significantly younger (mPHPT 42.6 ± 16.1 vs. sPHPT 63.2 ± 11.3 years *p* < 0.01) while in our study both groups were comparable in age).

Notably, the radius of BMD is much less frequently evaluated in MEN1. Lourenco DM et al. [6] demonstrated that in the younger MEN1 patients (<50 years of age), demineralization in the R33% was more frequent, more severe, and occurred earlier (40%; Z-score −1.81 ± 0.26) compared to the older group. In turn, the older group (>50 years of age) had a higher frequency of bone demineralization at all sites (*p* < 0.005) and had more severely altered BMD in the R33% (*p* = 0.007) and LS (*p* = 0.002). We did not find any differences in BMD in the R33% and RT (except for the Z-score in R33%) between the two studied groups, which could be due to the comparable age of the patients.

Excess PTH is thought to increase bone turnover, leading to bone loss, especially at cortical sites [43]. However, the use of high-resolution peripheral quantitative computed tomography (HRpQCT) confirmed deterioration in both trabecular and cortical bone in patients with PHPT [44]. Comparison of bone damage in mPHPT/sPHPT measured by HRpQCT did not reveal any differences [45]. In our study using 3D-DXA, mPHPT was strongly associated with more severe preoperative cortical thickness, cortical sBMD, and cortical vBMD deterioration than sPHPT. Trabecular vBMD was lower in FN (*p* = 0.008), but not in TH (*p* = 0.029); also we did not find differences in the TBS (RI > 1.310, *p* = 0.136). In contrast to our results, Song et al. [46] revealed a more severe trabecular bone involvement in mPHPT rather than in sPHPT, showing a statistical difference between groups in both LS (mPHPT 0.91 ± 0.18 vs. sPHPT 1.01 ± 0.17 g/cm^2^, *p* ≤ 0.001) and TBS (mPHPT 1.22 ± 0.14 vs. sPHPT 1.29 ± 0.11, RI > 1.310, *p* < 0.001). Presumably, these differences may be associated with longer disease duration: mPHPT 6.0 (3.0; 13.5) vs. sPHPT 2.0 (0.4; 6.0) years, *p* ≤ 0.001.

Data on bone recovery after PTE in mPHPT patients are extremely limited. Available studies are often small and retrospective, and the age of patients as well as the time intervals between postoperative DXA examinations vary widely. For example, in the study by Burgess et al. [47] only five patients with mPHPT had DXA data before and 1 year after primary PTE with a mean follow-up of 18 months [range 12–24 months]. Based on our results, the groups were comparable in terms of a postoperative DXA interval mPHPT of 1.2 [0.9; 1.3] vs. sPHPT 1.3 [1.1; 1.6] years, respectively, *p* = 0.267. PTE had a positive effect on bone recovery in both groups at the same sites: in the LS (mPHPT, *p* = 0.008; sPHPT, *p* = 0.005), FN (mPHPT, *p* = 0.007; sPHPT, *p* = 0.002), and TH (mPHPT, *p* = 0.005; sPHPT, *p* = 0.002). The absence of significant changes in BMD at RT and R33% may be related to structural characteristics of the radius. This bone, which acts as a lever, has a predominantly cortical rather than trabecular component, which is associated with lower bone turnover and high torsional resistance [48]. Thus, lack of mechanical loading, as a crucial factor in adult bone remodeling, can affect independently the process of BMD recovery at this site. The same findings were reported by Coutinho et al. [49]. They also showed the absence of significant changes in R33% and ultradistal radius in sixteen mPHPT patients from six families at 15 months after PTE. At the same time, significant increase was observed in other sites: in the LS (from 0.843 to 0.909 g/cm^2^; +8.4%, *p* = 0.001), FN (from 0.745 to 0.798 g/cm^2^; +7.7%, *p* = 0.0001), and TH (from 0.818 to 0.874 g/cm^2^; +6.9%, *p* ≤ 0.0001).

In our study, mPHPT patients showed statistically significant improvement in all of the main parameters of cortical and trabecular bone measured by 3D-DXA. However, the sPHPT group did not show the same results; there was no statistically significant increase in cortical thickness (TH *p* = 0.502; FN *p* = 0.855), cortical sBMD FN (*p* = 0.058), and trabecular vBMD TH (*p* = 0.035). The comparability of the groups with respect to the main DXA and 3D-DXA outcomes 1 year after PTE is unexpected, especially considering that mPHPT patients initially had lower preoperative BMD values than sporadic one. This demonstrates the reversibility of bone complications in both groups when remission is achieved. 3D-DXA could be used to better define the bone complications of mPHPT, especially due to the defined differences between the cortical and trabecular compartments. In addition, the data on the most affected compartment may help in the selection of antiosteoporotic therapy when surgery is contraindicated or not possible.

Obviously, the bone phenotype in mPHPT is different from the sporadic form of the disease. In addition to these multiple factors, the possible role of *MEN1* mutations in altering bone development and remodeling cannot be excluded [50].

## 5. Limitations and the Advantages of Research

The main limitations of our study are related to the retrospective design and the relatively small sample size due to the orphan nature of the disease and strict exclusion criteria. Vitamin D measurement was not routinely performed.

The advantages of the study are the comparability of the groups by sex and age, the comprehensive assessment of mineral metabolism and bone phenotype before and 1 year after radical surgery, and the presence of genetic testing in all participants to avoid potential misclassification of patients between groups. The additional influence of significant factors (endocrine and non-endocrine) on bone status was minimized by strict exclusion criteria for both groups, increasing the reliability of the results obtained. This is the first study to use 3D-visualization in mPHPT.

## 6. Conclusions

This is the first study with comparative analysis of calcium–phosphorus metabolism, DXA, and 3D-DXA in mPHPT and sPHPT patients. PHPT in MEN1 syndrome is associated with more severe preoperative BMD loss with predominant architecture disruption in the cortical bone. PTE positively affects BMD recovery in both the cortical and trabecular bone in mPHPT. The absence of difference in bone remodeling markers, DXA, and 3D DXA parameters 1 year after PTE between mPHPT/sPHPT combined with significantly lower preoperative BMD in the MEN1 group may indicate faster bone recovery in mPHPT. Further clinical and fundamental studies are required to identify the specificities of the bone phenotype in mPHPT. This is crucial for personalized treatment approaches in the future.

## Figures and Tables

**Table 1:** Characteristics of the calcium–phosphorus metabolism and PHPT complications before surgery.

		mPHPT		sPHPT	mPHPT vs. sPHPT	mPHPT vs. sPHPT
Variables	N	Me [Q1; Q3] or n (%)	N	Me [Q1; Q3] or n (%)	*p*-Value	*p*_0_-Value
PTH, pg/mL	22	131.60 [95.92; 198.30]	37	117.30 [102.30; 169.50]	0.931 ^1^	0.048
Albumin-corrected calcium (mmol/L)	22	2.69 [2.62; 2.80]	36	2.69 [2.63; 2.77]	0.911 ^1^	0.046
Phosphorus (mmol/L)	20	0.79 [0.71; 0.94]	36	0.81 [0.73; 0.89]	0.669 ^1^	0.045
eGFR mL/min/1.73 m^2^	22	110.30 [95.40; 116.70]	36	102.50 [93.30; 110.60]	0.194 ^1^	0.030
24-h urine calcium (mmol/L)	21	8.22 [6.42; 10.29]	37	8.62 [6.60; 10.90]	0.651 ^1^	0.043
Osteocalcin (ng/mL)	18	55.88 [34.32; 70.06]	29	48.66 [34.08; 61.16]	0.592 ^1^	0.038
b-CrossLaps (ng/mL)	17	1.00 [0.75; 1.26]	28	0.881 [0.61; 1.28]	0.665 ^1^	0.044
Nephrolithiasis	22	12 (54.5%)	37	23 (62.2%)	0.594 ^2^	0.039
Low-energy fractures	22	2 (9.1%)	37	2 (5.4%)	0.624 ^2^	0.040
Z-score less than −2.0 SD and/or low-energy fractures reported	22	13 (59.1%)	37	10 (27.0%)	0.026 ^2^	0.019

Abbreviations: PTH, parathyroid hormone; eGFR, estimated glomerular filtration rate. ^1^ Mann–Whitney test (U-test). ^2^ Fisher’s exact test (F-test).

**Table 2:** Summary of identified variants *MEN1*.

N	Sex, Age	HGVS Nomenclature	Protein	SNP	Type of Variant *	Significance	Reference
1	Male, 20	c.923C > A	p.Ser308Ter	rs1565644366	Nonsense	P	[14,15]
2	Female, 39	c.830_831insGTAC	p.Ala279TyrfsTer39	-	Frameshift	LP	Not described
3	Female, 18	c.1716_1718del	p.Ser573del	-	Frameshift	VUS	Not described
4	Female, 36	c.87_96del	p.Glu30ThrfsTer86	-	Frameshift	LP	Not described
5	Female, 29	c.1546dup	p.Arg516ProfsTer15	rs767319284	Frameshift	P	[15,16,17,18]
6	Male, 36	c.1340T > C	p.Phe447Ser	rs1941604532	Missense	P	[15,19]
7	Female, 34	c.87_96del	p.Glu30ThrfsTer86	-	Frameshift	P	Not described
8	Female, 37	c.398_436del	p.Tyr133_Ser145del	-	Frameshift	LP	Not described
9	Male, 22	c.1594G > T	p.Gly537Cys	rs587780843	Missense	VUS	[20]
10	Female, 41	c.658T > C	p.Trp220Arg	rs1085307971	Missense	LP	[21,22]
11	Female, 37	c.249_252del	p.Ile85Serfs *33	rs587776841	Frameshift	P	[23,24]
12	Female, 39	c.1252_1254delinsTAT	p.Asp418Tyr	-	Delins	P	Not described
13	Female, 32	c.784-9G > A	-	rs794728625	Splicing	P	[25,26,27,28,29,30]
14	Female, 36	c.1252G > T	p.Asp418Tyr	rs104894264	Missense	P	[31]
15	Female, 35	c.1546dup	p.Arg516Profs * 15	rs767319284	Frameshift	P	[15,16,17,18]
16	Female, 40	c.-23-14G > A	-	rs886048479	Splicing	VUS	Not described
17	Female, 28	c.681C > T	p.Tyr227	rs778921501	Synonymous	LB	Not described
18	Female,40	c.658T > C	p.Trp220Arg	rs1085307971	Missense	LP	[21,22]
19	Female, 44	c.467G > A	p.Gly156Asp	rs794728648	Missense	P	[32,33,34]
20	Female, 25	c.658T>C	p.Trp220Arg	rs1085307971	Missense	LP	[21,22]
21	Female, 39	c.772C > T	p.Gln258Ter	rs886039416	Nonsense	P	[14,35,36]
22	Male, 24	c.1602_1618dup	p.Pro540ArgfsTer25	rs794728660	Frameshift	P	[37]

All variants described are based on Reference Sequence NM_001370259.2 (NP_001357188.2) *—https://hgvs-nomenclature.org/stable/, accessed on 1 September 2024. SNP—single nucleotide polymorphism. P—Pathogenic, LP—Likely-pathogenic; VUS—Variant of unknown significance; LB—Likely benign.

**Table 3:** Comparisons of the DXA and 3D-DXA parameters between mPHPT and sPHPT groups before surgery.

		mPHPT		sPHPT	mPHPT vs. sPHPT	mPHPT vs. sPHPT
Variables	N	Me [Q1; Q3]	N	Me [Q1; Q3]	*p*-value	*p*_0_-value
BMD LS g/cm^2^	21	1.02 [0.93; 1.11]	37	1.15 [1.07; 1.22]	**0.002**	0.006
LS Z-score	21	−1.50 [−1.90; −1.00]	37	−0.50 [−1.20; −0.10]	**0.012**	0.016
BMD FN g/cm^2^	22	0.81 [0.67; 0.94]	37	0.94 [0.88; 1.04]	**0.001**	0.004
FN Z-score	22	−1.60 [−1.90; −0.80]	37	−0.40 [−1.0; 0.00]	**0.004**	0.011
BMD TH g/cm^2^	22	0.89 [0.72; 0.92]	37	0.97 [0.89; 1.10]	**0.002**	0.008
TH Z-score	22	−1.00 [−1.80; −0.40]	36	−0.40 [−0.90; 0.40]	0.018	0.018
BMD RT g/cm^2^	22	0.58 [0.53; 0.66]	35	0.64 [0.58; 0.72]	0.059	0.023
RT Z-score	22	−1.50 [−2.60; −0.30]	36	−0.60 [−1.60; 0.30]	0.062	0.024
BMD R33% g/cm^2^	22	0.74 [0.68; 0.85]	35	0.82 [0.78; 0.89]	0.036	0.021
R33% Z-score	21	−1.50 [−2.3; −0.9]	15	−0.60 [−1.10; 0.00]	**0.007**	0.013
TBS (RI > 1310)	9	1.39 [1.32; 1.45]	37	1.49 [1.40; 1.51]	0.136	0.029
Cortical sBMD TH (mg/cm^2^)	22	131.15 [106.96; 150.63]	37	151.95 [141.89; 163.72]	**0.001**	0.005
Cortical sBMD FN (mg/cm^2^)	22	102.06 [92.54; 118.58]	37	130.10 [119.68; 138.09]	**<0.001**	0.001
Trabecular vBMD TH (mg/cm^3^)	22	142.22 [105.29; 181.17]	37	168.81 [150.22; 212.23]	0.029	0.020
Trabecular vBMD FN (mg/cm^3^)	22	181.93 [154.69; 235.27]	37	237.74 [212.92; 265.67]	**0.008**	0.015
Cortical vBMD TH (mg/cm^3^)	22	724.79 [652.67; 779.78]	37	800.74 [751.19; 857.710]	**0.007**	0.014
Cortical vBMD FN (mg/cm^3^)	22	713.81 [671.471; 768.502]	37	797.82 [758.03; 858.38]	**0.002**	0.009
Cortical Thickness TH (mm)	22	1.77 [1.65; 1.83]	37	1.910 [1.86; 2.01]	**<0.001**	0.003
Cortical Thickness FN (mm)	22	1.48 [1.40; 1.59]	37	1.65 [1.49; 1.80]	**0.002**	0.010

Abbreviations: BMD, bone mineral density; LS, lumbar spine; FN, femur neck; TH, total hip; RT, radius total; R33%, distal third of the radius; TBS, trabecular bone score; RI—reference interval; sBMD, surface bone mineral density; vBMD, volumetric bone mineral density. The Mann–Whitney test (U-test) was used to compare the indicators. Statistically significant differences are shown in bold (*p*-value < *p*_0_-value).

**Table 4:** 3D-DXA parameters before and after surgery in mPHPT.

		mPHPT (Before Surgery)		mPHPT (After Surgery)	mPHPT Before and After Surgery	mPHPT Before and After Surgery
Variables	N	Me [Q1; Q3]	N	Me [Q1; Q3]	*p*-Value	*p*_0_-Value
Cortical sBMD TH (mg/cm^2^)	11	135.70 [100.65; 153.83]	11	147.71 [106.21; 168.08]	**0.001**	0.004
Cortical sBMD FN (mg/cm^2^)	11	112.20 [95.04; 123.62]	11	121.33 [101.16; 132.55]	**0.001**	0.008
Trabecular vBMD TH (mg/cm^3^)	11	157.17 [113.95; 177.26]	11	172.62 [120.75; 226.64]	**0.019**	0.035
Trabecular vBMD FN (mg/cm^3^)	11	204.18 [170.26; 226.75]	11	207.64 [170.02; 286.12]	**0.019**	0.038
Cortical vBMD TH (mg/cm^3^)	11	745.44 [597.69; 776.26]	11	761.41 [614.26; 835.44]	**0.005**	0.012
Cortical vBMD FN (mg/cm^3^)	11	738.11 [636.25; 781.54]	11	767.69 [667.84; 816.35]	**0.019**	0.042
Cortical Thickness TH (mm)	11	1.79 [1.68; 1.96]	11	1.878 [1.73; 2.01]	**0.005**	0.015
Cortical Thickness FN (mm)	11	1.61 [1.50; 1.71]	11	1.65 [1.58; 1.71]	**0.007**	0.023

Abbreviations: sBMD, surface bone mineral density; vBMD, volumetric bone mineral density. Note. The Wilcoxon test (W-test) was used to compare two dependent groups for quantitative characteristics (*p*-value). Statistically significant differences are shown in bold (*p*-value < *p*_0_-value).

**Table 5:** 3D-DXA parameters before and after surgery in sPHPT group.

		sPHPT (Before Surgery)		sPHPT (After Surgery)	sPHPT Before and After Surgery	sPHPT Before and After Surgery
Variables	N	Me [Q1; Q3]	N	Me [Q1; Q3]	*p*-value	*p*_0_-value
Cortical sBMD TH (mg/cm^2^)	14	157.06 [140.41; 178.59]	14	174.11 [147.50; 186.59]	**0** **.005**	0.012
Cortical sBMD FN (mg/cm^2^)	14	133.07 [123.91; 149.90]	14	142.11 [131.30; 150.76]	0.058	0.035
Trabecular vBMD TH (mg/cm^3^)	14	166.93 [144.94; 232.28]	14	203.19 [151.75; 229.46]	0.035	0.031
Trabecular vBMD FN (mg/cm^3^)	14	232.96 [213.04; 287.85]	14	251.00 [224.43; 278.63]	**0.020**	0.027
Cortical vBMD TH (mg/cm^3^)	14	816.77 [766.93; 935.14]	14	843.29 [835.00; 925.19]	**0.013**	0.023
Cortical vBMD FN (mg/cm^3^)	14	826.03 [788.36; 910.54]	14	845.87 [819.88; 905.26]	**0.011**	0.019
Cortical Thickness TH (mm)	14	1.93 [1.85; 2.11]	14	1.98 [1.87; 2.10]	0.502	0.042
Cortical Thickness FN (mm)	14	1.70 [1.57; 1.84]	14	1.67 [1.55; 1.84]	0.855	0.050

Abbreviations: sBMD, surface bone mineral density; vBMD, volumetric bone mineral density. Note. The Wilcoxon test (W-test) was used to compare two dependent groups for quantitative characteristics (*p*-value). Statistically significant differences are shown in bold (*p*-value < *p*_0_-value).

## Data Availability

The original contributions presented in this study are included in the article, further inquiries can be directed to the corresponding author.
